# A New Atlas Localization Approach for Subthalamic Nucleus Utilizing Chinese Visible Human Head Datasets

**DOI:** 10.1371/journal.pone.0057264

**Published:** 2013-02-27

**Authors:** Jingjing Rong, Qinghua Wang, Kaijun Liu, Liwen Tan, Xu Ran, Shaoxiang Zhang, Qiyu Li, Yaling Han

**Affiliations:** 1 Chongqing Institute of Computing Medicine, Department of Anatomy, College of Basic Medical Sciences, Third Military Medical University, Chongqing, China; 2 Department of Neurology and Centre for Clinical Neuroscience, Daping Hospital, Third Military Medical University, Chongqing, China; 3 Network Information Center, Training Department, Chongqing Communication College, Chongqing, China; 4 Department of Cardiology, Shenyang Military General Hospital, Shenyang, China; Universität München, Germany

## Abstract

**Objective:**

To study the possibility of Chinese visible human (CVH) head datasets as brain atlas for locating the subthalamic nucleus (STN) before deep brain stimulation (DBS) surgery.

**Methods:**

Optimal head axial and coronal 3.0T, T2-weighted magnetic resonance images (MRI) of 30 patients were obtained. Cross-sectional head images of four CVH head datasets were chosen to establish an average CVH model. All MRI sequences were registered to the CVH model via fiducials in X-, Y-, and Z-direction, respectively, within the same stereotactic space. The correlations between red nucleus (RN) and fiducials, the accuracy of MRI-to-CVH registration, and the coordinate value differences of RN gravity center between MRI and CVH were tested.

**Results:**

The mean gravity center coordinate values and ranges of STN in CVH were measured; The X coordinate value of RN positively correlates with the most anterior point of putamen(PU-A), the lateral edges of putamens (PU-L), and width of the third ventricle, negatively correlated with the patients’ age; The minimal mean errors were when no fiducials were used when locating RN between the MRI and the CVH average model; There were no significant differences of RN in X- and Y-direction between MRI sequences and CVH.

**Conclusion:**

CVH head datasets can be used as brain atlas for Chinese STN localization. Moreover, average coordinate values of the gravity center and the ranges of STN in CVH can be considered as references for locating STN.

## Introduction

The subthalamic nucleus(STN) is a biconvex, lens-shaped nucleus located in the subthalamus of the diencephalon. It lies medial to the internal capsule and immediately rostral to the level at which the latter becomes continuous with the crus cerebri of the midbrain. The STN plays a central role in the normal function of the basal ganglia and in the pathophysiology of basal ganglia-related disorders. The subthalamic nucleus is also crucially involved in the pathophysiology of Parkinson’s disease (PD) and is a target of functional neurosurgical therapy for that condition [1]. Deep brain stimulation of the subthalamic nucleus (STN-DBS) has turned out to be an optimal surgical treatment option for advanced or refractory PD patients, and the precision of stimulus location in the STN is extremely important to assure maximal therapeutic benefit and the fewest side effects [2], [3], [4], [5].

Two approaches are used to identify the stimulating site and reduce misplacement before electrodes are implanted in most DBS centers, including measuring stereotactic coordinate values of the stimulating site in STN according to a patient’s MRI directly and/or utilizing the relationship of STN to the brain midcommissural point from a stereotactic brain atlas indirectly. The higher precision of target coordinate values before operation, the smaller number of microelectrode recording will be performed and the lower chance of intracranial hemorrhage will be exist [6].

Magnetic resonance imaging (MRI) is currently considered as the most powerful imaging technique to display brain structures in vivo. However, visualization of STN on MRIs is still difficult. One reason is that the hypointensity signal of STN from anterior to posterior is inhomogeneous [7], [8], [9]. Another reason is that the spatial resolution of MRI is not sufficient for precisely imaging of STN. The dorsolateral or anterior lateral and superior portions of STN exhibits an undetectable contrast and thus remains poorly visible on MRIs, which are most frequently used as target territory (called sensorimotor territory) for STN-DBS in PD [10], [11], [12], [13], [14]. Therefore, the precision of positioning the stimulus location on a MRI directly is questionable.

To solve these problems, some investigators suggest using atlas-based scaling to improve STN localization. The Schaltenbrand and Wahren Atlas for Stereotaxy of the Human Brain [15] has been widely used to locate STN as an indirect method. Yelnik et al. [4] attempted a three-dimensional Schaltenbrand and Wahren atlas to visually match MR image with a corresponding atlas slices. However, this atlas is based on three different cerebral hemispheres for each series of sections (axial, coronal, and sagittal). The coordinates of STN in the Schaltenbrand and Wahren atlas are thus accurate for only a subgroup of the PD population [6].

The Mai atlas [16] consists of coronal stained histological sections at 1.34 mm intervals and was chosen as a reference to identify the boundary of STN in MRI [3]. The combined three-dimension histological and MR images atlas localization approach, created by Yelnik et al. [17], [18], should likely be an improvement over the existing approaches based on the published atlases because of its availability in a digital format. Yet as we know, there is no atlas brain that will not deform and distort more or less during the manufacturing operation, including CSF leaking which results in ventricle collapse during brain extraction; tissue shrinkage in fixation, dehydration and freezing; and distortion during section slicing, mounting and staining, etc. These potential problems exist in any atlas based on the study of post mortem brains [3]. In addition, there are some morphological differences of deep brain structures between the Chinese and White brains [19]. And the applicability of atlases based on White brains to PD patients in China needs further research.

To improve the atlas-based localization method, the cross-sectional head images from four Chinese visible human (CVH) datasets [20] are chosen in our research. The cross-sectional head images are of real color, high resolution, regular thin intervals, and no deformation. Furthermore, they contain integrated morphological information on all structures in the head and permit more accurate recognition and position of intracranial structures whose contours are indistinct in MRI.

In this research, the feasibility of CVH head datasets for STN localization was studied as brain atlas. All the defined fiducials in both MRI and CVH head datasets, which represent the general and local morphology of cerebrum and midbrain to some extent, are close to or bound to the spatial area containing STN. Therefore, they are expected to improve STN localization in the deep brain.

## Materials and Methods

### MRI Sequence Acquisition and Processing

**Figure 1:**
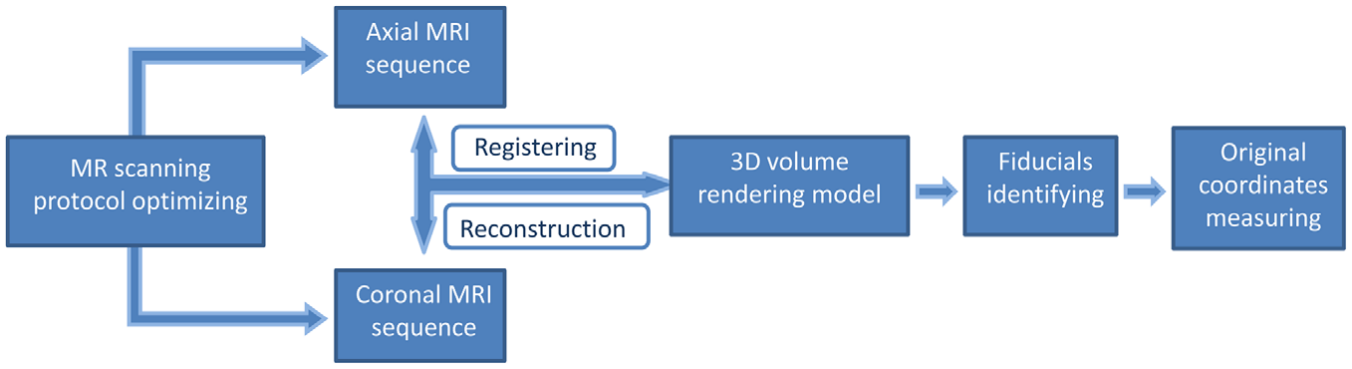
Flowchart of successive steps of MRI acquisition and processing. The successive steps are scanning protocol optimizing, MRI acquisition, homogenous axial and coronal MR sequences registering, three-dimensional volume rendering reconstruction, fiducials identifying, and original coordinates of fiducials measuring.

The successive steps of MRI acquisition and processing, comprising scanning protocol optimizing, image acquisition, homogenous axial and coronal MRI sequences registratioin, three-dimensional (3D) volume rendering reconstruction, fiducials identifying, and original coordinates of fiducials measuring, were presented in Figure 1.

#### Acquisitions of MRIs

Cranial MRI sequences for 30 patients (7 males and 23 females) without intracranial organic lesions were included in this study. These patients, whose age ranged from 27 to 65 years (mean 48.2, SD 10.55), were scanned as part of their routine clinical examinations to rule out brain disease. MRIs were acquired with a SIEMENS Magnetom Verio 3.0T scanner and included two T2-weighted turbo spin echo sequences. One was acquired in axial planes (parallel to the AC-PC plane) covering the whole brain and another was acquired in coronal planes, from a few centimeters ahead of the knee of the corpus callosum to the posterior of the cerebellum. The T2-weight MR scanning protocol was optimized(orientation-axial, TR 5220 ms, TE 96 ms, flip angle 146°, slice thickness 2 mm/0 gap, FOV 230 mm, matrix 512×512 pixels, averages 3; orientation-coronal, TR 9560 ms, TE 97 ms, flip angle 120°, slice thickness 2 mm/0 gap, FOV 230 mm, matrix 256×256 pixels, averages 7). A fixed device was used to eliminate noticeable movement of the head during scanning, and accordingly, the MRIs were free from any quality-limiting motion artifact.

#### Fiducial identification of the MRIs

**Figure 2:**
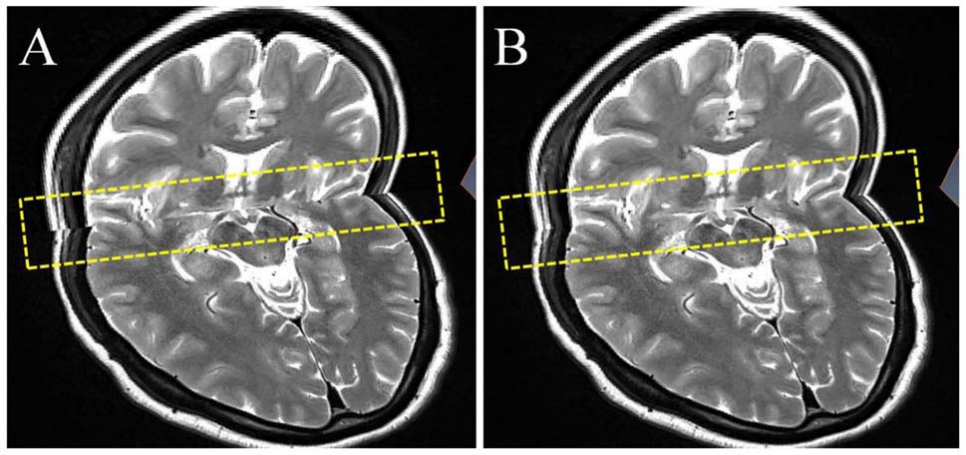
Homogenous axial and coronal MR sequences before and after registering in Amira software. The intersection area of axial and coronal images is shown by dotted boxes. (A) Before registering, the spatial mismatches between axial and coronal MR sequences are quite obvious. (B) After registering, the axial MR sequence matches very closely with the coronal MR sequence.

The homogenous axial and coronal MRI sequences of each patient were simultaneously imported into the SGI graphics workstation equipped with Amira software. When the MRI sequence was transferred into the Amria software, the upper-left corner pixel in the first image was automatically defined as the origin of the 3D space of the whole image sequence and the right side, posterior, and inferior to the origin were defined as positive. Then all the pixels in MRI sequences were given the default space coordinates at the same time, named original coordinate values. Then, axial and coronal MRI sequences were registered via an automatic registration algorithm to obtain the correct spatial corresponding relationships between them (Figure 2). The registration module of Amria software provided an automatic registration for homogenous images sequences via optimization of quality function, and so a hierarchical strategy of rigid-registration was applied, starting with a coarse resampling of the datasets and proceeding to finer resolutions later. After rigid-registration, a visual 3D volume rendering model was reconstructed based on homogenous registered MRI sequences in Amira software and the defined structural fiducials, which was close or adjacent to STN and RN, were identified in the registered axial and coronal MRI in 3D model. Then, the original coordinate values of fiducials were measured, including the centers of anterior commissure (AC) and posterior commissure (PC), the anterior tip of biputamen(PU-A),the lateral tip of biputamen(PU-L), the anterior tip of optic chiasm(OX) center, and the gravity centers of both sides red nuclei(RN) for later coordinate transformation.

For AC, anterior-posterior (AP),lateral-medial (LM) and superior-inferior (SI) locations were identified as the middle of the dark line in corresponding axial and coronal planes. PC was a dark and narrow line at the back of the third ventricle directly in the axial plane, while the middle of the dark line was the AP and LM location of the PC. For the anterior tip of putamen (PU-A), the AP, LM, and SI locations were identified as the most anterior tip of putamen, emerged in the anterior commissure and the posterior commissure(AC-PC) axial plane. The lateral edges of putamens(PU-L) were in the AC-PC axial plane where the coronal mid-commissures plane intersected. The OX location was obtained by a careful review of consecutive coronal images on which the optic nerve, optic chiasm, and optic tract could be found. The AP location was identified on the coronal plane, where OX appeared first and at the middle of the thickest; the darkest horizontal narrow band was considered as the SI location. The LM location was set as the crossing point for the first coronal plane, including OX and the mid-sagittal plane (dividing the third ventricle equally). For RN, AP LM, and SI locations were acquired to estimate the gravity center of RN by choosing the axial and coronal plane that displayed the largest circle representing RN and locating the point in the center of the circle. The width of the third ventricle (3rd-V) was measured at the intersecting line of the AC-PC axial plane and the mid-commissures plane. All fiducials in the MRI T2 sequences were located on corresponding images using red cursors with 0.03 mm precision in Amira software.

### CVH Image Acquisition and Processing

**Figure 3:**
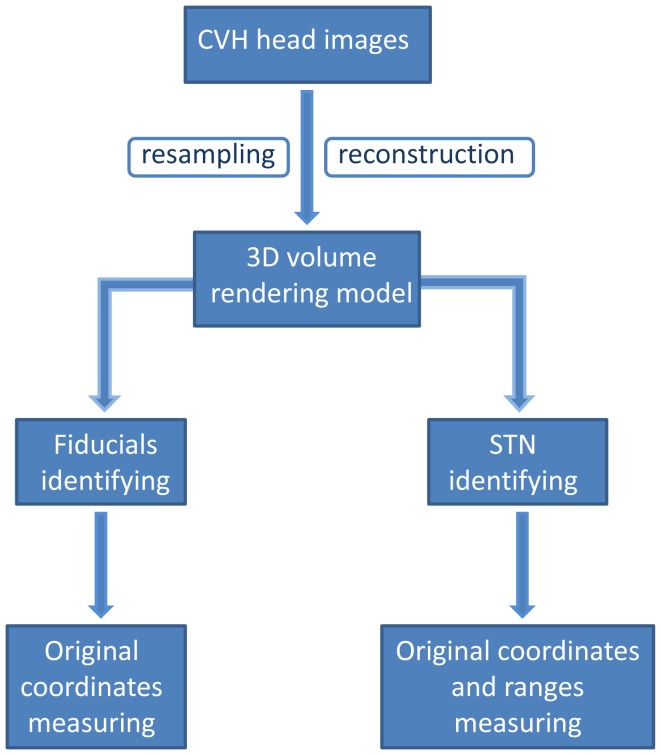
Flowchart of CVH head image acquisition and processing. The successive steps are CVH-1, 2, 4, 5 head image acquisition, resampling, three-dimensional volume rendering reconstruction, fiducials and subthalamic nucleus (STN) identifying, original coordinates of fiducials measuring, and original ranges and gravity center coordinates of STN measuring.

The successive steps of CVH images processing, comprising acquisition, resampling, 3D volume rendering reconstruction, fiducials identifying, original coordinates of fiducials measuring, identifying STN, and measuring the original coordinates and ranges of STN were presented in Figure 3.

#### Acquisition of CVH images

Successive thin-layer, cross-sectional head images, including the whole cerebrum and cerebellum, were retrieved from four different CVH datasets (CVH-1, 2, 4, 5). Heads of the four cadavers, without organic lesions, were milled transversely under a low temperature environment from the Reid’s-base line. All the cross-sectional images of heads were photographed using a digital camera in fixed position. General information on the four cadaver specimens and related parameters of cross-sectional images were shown in Table 1.

**Table 1:** General information of the four CVH cadaver specimens and the cross-sectional images parameters of the four CVH datasets.

	CVH-1	CVH-2	CVH-4	CVH-5
**Age**	35	22	25	25
**Gender**	Male	Female	Female	Female
**Height(cm)**	170	162	162	170
**Weight(kg)**	65	54	57.7	59
**Slice interval(mm)**	0.5	0.25	0.25	0.2
**Images resolution(pixel)**	3072×2048	3072×2048	4064×2704	4064×2704
**Each image size(MB)**	18	18	31.5	31.5

#### Fiducials and STN identification of CVH images

We imported image sequences of four CVH head datasets into Amira software in succession and then rotated cross-sectional image sequences at certain angles to meet the mid-sagittal plane and adjusted the axial plane of the images to the AC-PC axial plane by resampling the datasets as well. Then the coronal and sagittal planes were automatically generated. After rotation and resampling, the orientations of three new orthogonal planes of the four CVH head datasets were parallel to the MRI. Fiducial identification and coordinate value obtained were treated in the same way as the MRI sequences.

The STN is a biconvex lens-shaped nucleus in the deep brain and it is spindle in the orthogonal planes. In order to accurately identify the ranges and gravity center of STN in CVH-1, 2, 4, 5, we tracked the outlines of STN in axial, coronal and sagittal images continuously. The intersection point of orthogonal planes, which displayed the largest area of STN in axial-, coronal- and sagittal-direction, was considered as the gravity center. And then, the ranges and gravity center coordinate values of STN in the original coordinate system were measured by Amria software.

**Figure 4:**
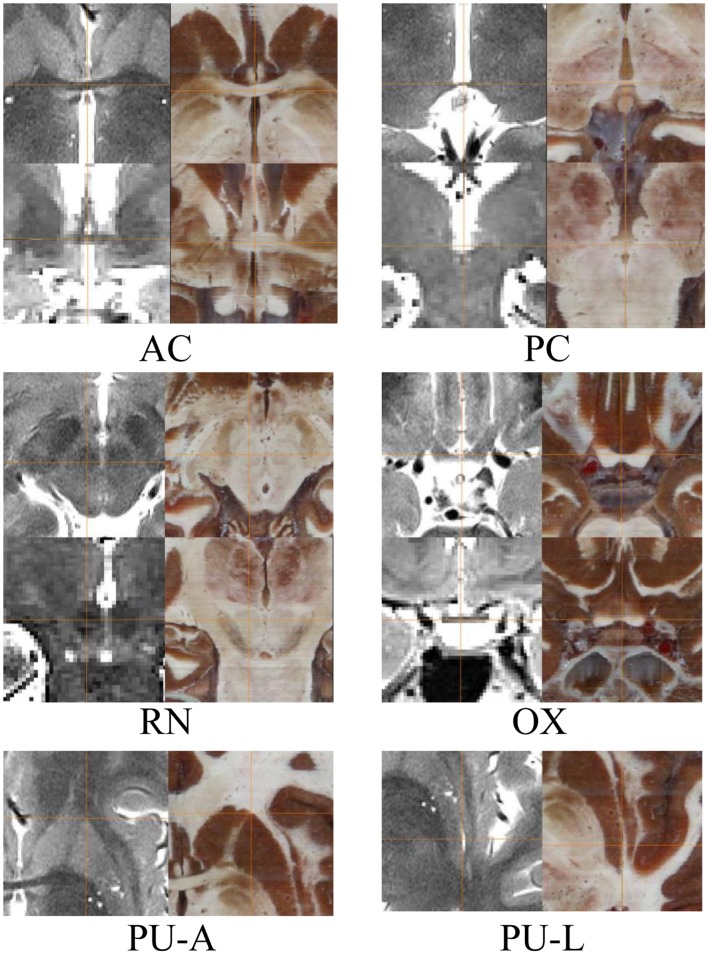
Cross lines identify the locations of all fiducials in the MRI (left) and CVH (right). For AC, PC, OX and RN, the left-side images are the axial (above) and coronal (below) section of T2-weighted MRIs; the right-side images are axial (above) and coronal (below) section of CVH datasets. Two axial sections are shown for PU-A and PU-L, respectively. AC: Center of anterior commissure; PC: Center of posterior commissure; RN: Gravity center of red nucleus; OX: Anterior tip of optic chiasm center; PU-A: Anterior tip of putamen; PU-L: Lateral tip of putamen.

**Figure 5:**
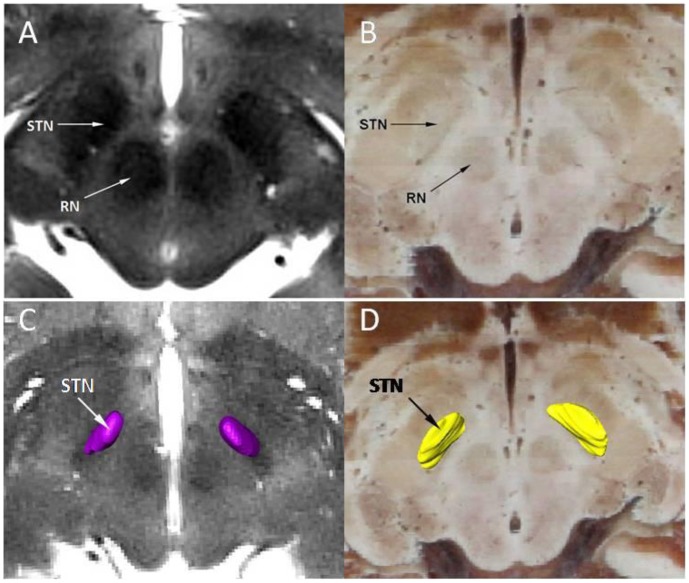
The 2D morphology of STN and 3D surface rendering reconstruction models of STN. The 2D morphology of STN and RN in MRI and CVH are shown in A and B; the 3D surface rendering reconstruction models of STN, based on MR sequences (C) and CVH successive cross-sectional images (D), are displayed. RN: Red nucleus; STN: Subthalamic nucleus.

Ascribing to real color and high resolution, recognizing anatomical structures was easier, and thus it became more accurate to identify the fiducials and STN in the CVH images than in the MRI images. The fiducial identification in the MRI and CVH images are illuminated in Figure 4. The RN and STN in two-dimensional (2D) MRIs and CVH images were shown(Figure 5A and 5B), as well as the 3D surface rendering reconstruction models of STN based on MR sequences and CVH successive cross-sectional images in Amira software (Figure 5C and 5D). Visualization of STN on 2D MRIs was difficult (Figure 5A), but it was easier on CVH images (Figure 5B). The 3D reconstruction model of STN in MRI sequence, based on threshold segmentation, could not truly display the whole 3D shape of STN (Figure 5C) because of the undetectable contrast and unidentified outline of STN, while the CVH datasets showed good results (Figure 5D).

### Establishment of a Head Space Coordinate System

**Figure 6:**
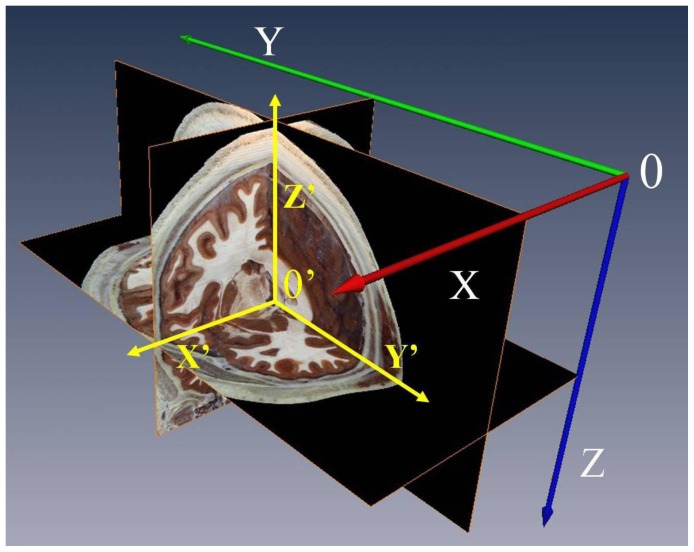
The original and new 3D coordinate system of head space. In the original 3D coordinate system, the upper-left corner pixel (0) in the first image is defined as the origin of the three dimensional space of the whole images sequence and the right side(X), posterior(Y), and inferior (Z) to the origin were defined as positive. In the new 3D coordinate system, the midpoint of the anterior commissure and the posterior commissure (AC-PC) is defined as the origin (0′), and the right side(X’), anterior(Y’), and superior(Z’) to the origin are defined as positive.

A new 3D coordinate system of head space was established here (Figure 6). We took the midpoint of AC-PC as the origin (0 mm, 0 mm, 0 mm), and the anterior, superior and right side to the origin were defined as positive. Original coordinate values of the fiducials in 30 MRI sequences and in CVH-1, 2, 4, 5 head datasets were transformed into the new coordinate system respectively, as well as the ranges and the gravity center coordinates of STN in CVH-1, 2, 4, 5 (Table 2).

**Table 2:** Ranges/gravity center coordinates of subthalamic nucleus (STN) in CVH head datasets.

	CVH-1	CVH-2	CVH-4	CVH-5	CVH
**STN-left-X**	−15.57∼–5.52/−10.7	−13.51∼–6.45/−9.5	−13.72∼–4.23/−10.0	−12.61∼–3.64/−8.3	−13.85∼−4.96/−9.63
**STN-left -Y**	−5.19∼4.02/−1.6	−5.07∼2.61/−1.4	−6.54∼3.97/−2.0	−5.07∼6.37/0.8	−5.47∼4.24/−1.05
**STN-left- Z**	−8.04∼–2.01/−5.4	−6.91∼–0.77/−3.3	−6.79∼–0.64/−3.6	−7.15∼0.39/−3.5	−7.22∼–0.76/−3.95
**STN-right-X**	5.36∼15.07/10.0	6.6∼13.66/9.5	5.13∼13.97/10.3	4.42∼12.87/9.5	5.38∼13.89/9.83
**STN-right -Y**	−4.35∼4.69/−0.1	−5.07∼2.61/−1.0	−6.03∼4.23/−1.2	−5.46∼6.11/−0.3	−5.23∼4.41/−0.65
**STN-right- Z**	−8.04∼–2.01/−4.9	−6.97∼−0.77/−3.4	−6.28∼–0.13/−3.2	−7.54∼0.26/−3.5	−7.19∼−0.66/−3.75

All measurements are in mm.

### Establishment of an Average Model of CVH-1, 2, 4, 5

We calculated the corresponding mean values of the fiducials, and the ranges and gravity center coordinate values of STN in CVH-1, 2, 4, 5 as an average model (named CVH) for later analyses: AC (0 mm, 11.85 mm, 0 mm), PC(0 mm, −11.85 mm, 0 mm); PU-A-right (15.63 mm, 28.78 mm, 0 mm); PU-A-left (−15.80 mm, 28.38 mm, 0 mm); PU-L-right (28.40 mm, 0 mm, 0 mm); PU-L-left (−28.68 mm, 0 mm, 0 mm); OX(0 mm, 18.60 mm, −17.20 mm); RN-right(4.43 mm, −5.90 mm, −6.40 mm); and RN-left(−4.65 mm, −5.93 mm, −6.38 mm).

### Statistical Analysis

Data analyses were performed using SPSS software. All the coordinates of fiducials and red nuclei (RN) in 30 MRI sequences were evaluated for normal distribution with a K-S test.

#### Correlations of RN and fiducials in 30 MRI sequences

The correlations among the coordinates of RN and fiducials (including the age) in 30 MRI sequences were measured using Pearson coefficient (Table 3). The X, Y, Z coordinates of 60 RNs, X coordinates of 60 PU-As and 60 PU-Ls, Y and Z coordinates of 30 OXs, 30 AC-PC lengths, 30 values of third ventricle width (3rd-V) and age are included.

**Table 3:** Correlations between red nucleus (RN) and fiducials in 30 MRI sequences.

	PU-A-X	PU-A-Y	OX-Y	OX-Z	AC-PC	PU-L-X	3rd-V	age
RN-x	0.433^**^	0.266	−0.197	−0.058	0.229	0.546**	0.404*	−0.415*
RN-Y	0.125	−0.014	−0.268	0.118	−0.114	0.078	0.181	−0.207
RN-Z	0.327	0.221	−0.143	0.146	−0.134	0.401	0.257	0.02

* p<0.05; **: p<0.001.

RN: Gravity center of red nucleus; PU-A: Anterior tip of putamen; OX: Anterior tip of optic chiasm center; AC-PC: Distance between centers of anterior commissure and posterior commissure; PU-L: Lateral tip of putamen; 3rd-V: third ventricle.

#### 3D Coordinates space registration of MRI-to-CVH

Registering MRI sequences to CVH utilized seven fiducials to define the stretch ratio of gravity center coordinates of RN. The fiducials were used to define distances along X-, Y-, and Z-direction. The coordinate values ratios of MRI fiducals to corresponding fiducals in CVH were calculated as stretch ratios respectively. For X-direction stretch, PU-A, PU-L and no stretch were tested to determine the most accurate fit. For Y-direction stretch, AC-PC was tested and also no stretch. For Z-direction, OX and no stretch were tested. Then, the coordinate values of RN in MRI were scaled, according to the stretch ratios of fiducals in X-, Y-, Z-directions, for meeting the goal of registration of MRI-to-CVH. Finally, the difference of coordinate values of RN between registered MRI sequences and CVH were calculated (Table 4).

**Table 4:** Difference in localization of the gravity center of red nucleus (RN) using fiducials-based MRI-to-CVH registration.

	X			Y		Z		X			Y		Z	
Fiducials used	PU-A-r	PU-L-r	Non	AC-PC	Non	OX	Non	PU-A-l	PU-L-l	Non	AC-PC	Non	OX	Non
Mean deviation	0.63	0.38	0.32*	0.54	0.51*	1.33	1.07*	0.88	0.51	0.38*	0.52	0.49*	1.33	1.07*
Standard deviation	0.34	0.24*	0.39	0.36*	0.37	0.97	0.93*	0.41	0.29*	0.29*	0.39	0.37*	0.97	0.93*
Maximum deviation	1.08	1.02*	1.69	1.4*	1.46	2.94	2.40*	1.69	1.03	0.85*	1.56*	1.62	2.94	2.40*

* The minimum in the same group.

All values are in mm.

AC: Center of anterior commissure; PC: Center of posterior commissure; RN: Gravity center of red nucleus; OX: Anterior tip of optic chiasm center;PU-A: Anterior tip of putamen; PU-L: Lateral tip of putamen.

#### Coordinate value differences of RN and fiducials between MRI and CVH

Ranges and mean coordinate values of RN and fiducials in X-, Y-, Z-direction in 30 MRI are calculated, as well as the differences of the corresponding values in MRI and in CVH (Table 5).

**Table 5:** Ranges and mean coordinate values of RNs and fiducials in 30 MRI sequences and the differences in mean coordinate values between MRI and CVH.

	Range inMRI	Mean(SD)in MRI	Meanin CVH	P^a^
RN-X(right)	5.59–3.87	4.58(0.49)	4.43	NS
RN-Y(right)	−7.41–−5.40	−6.01(0.63)	−5.9	NS
RN-Z(right)	−7.00– −4.40	−5.46(1.06)	−6.4	0<0.001
RN-X(left)	−5.40– −3.60	−4.46(0.48)	−4.65	NS
RN-Y(left)	−7.53– −5.40	−6.06(0.61)	−5.93	NS
RN-Z(left)	−7.20– −4.20	−5.46(1.06)	−6.38	0<0.001
AC	13.05–11.65	12.26(0.39)	11.85	0<0.001
PC	−13.05– −11.65	−12.26(0.39)	−11.85	0<0.001
PU-A-X(right)	21.49–14.82	18.88(1.83)	15.63	0<0.001
PU-A-X(left)	−22.00– −16.95	−19.21(1.41)	−15.80	0<0.001
PU-L-X(right)	34.66–28.30	31.19(1.66)	28.40	0<0.001
PU-L-X(left)	−34.76– −28.34	−31.35(1.74)	−28.68	0<0.001
OX-Y	22.69–15.93	19.22(1.58)	18.60	NS
OX-Z	−19.40– −13.4	−16.36(1.47)	−17.20	0<0.001
Length of AC-PC	26.10–23.30	24.52(0.78)	23.68	0<0.001
Width of 3rd-V	5.00–0.90	2.88(1.12)	0.99	0<0.001

a Independent samples t test; NS: not significant(P>0.05);All values are in mm.

RN: Gravity center of red nucleus; AC: Center of anterior commissure; PC: Center of posterior commissure; PU-A: Anterior tip of putamen; PU-L: Lateral tip of putamen; OX: Anterior tip of optic chiasm center; 3rd-V: third ventricle.

## Results

### Average Position of STN in CVH Head Datasets

The average coordinate ranges of right and left STN in CVH-1, 2, 4, 5 were X(5.38 mm∼13.89 mm); Y(−5.23 mm∼4.41 mm); Z(−7.19 mm∼−0.66 mm) and X(−13.85 mm∼−4.96 mm); Y(−5.47 mm∼4.24 mm); Z(−7.22 mm∼−0.76 mm). The average coordinates values of the gravity centers of right and left STN in CVH-1,2,4,5 were (9.83 mm, −0.65 mm, −3.75 mm) with SD(0.69 mm, 0.92 mm, 1.34 mm) and (−9.63 mm, −1.05 mm, −3.95 mm) with SD(1.62 mm, 2.18 mm, 1.69 mm)(Table 2).

### Correlations between RN and Fiducials in MRI Sequences

Correlations between the coordinate values for red nucleus (RN) and fiducials in 30 MRI (including the age) are shown in Table 3. The X coordinate value of the RN gravity center positively correlates with the PU-A (P<0.001), the PU-L (P<0.001) and the width of the third ventricle (P<0.05), and negatively correlated with the patients’ age (P<0.05). The coordinates of the RN gravity center are totally unrelated to the AC-PC and OX in Y-direction, and OX in Z-direction as well as the patients’ ages.

### Results for MRI-to-CVH Registration

There were certain differences between the registered MRI sequences and CVH in terms of accuracy for all the fuducials (Table 4). In the X-direction, minimal mean errors were 0.32 mm(right) and 0.38 mm(left) with SD of 0.39 mm and 0.29 mm when no fiducials was used, and a maximum of 0.77 mm(right) and 1.15 mm(left) occurring when PU-A was used. In the Y-direction, minimal mean errors were 0.51 mm(right) and 0.49 mm(left) with the same SD of 0.37 mm in both sides when no fiducials was used compared with 0.54 mm(right) and 0.52 mm(left) with SD of 0.36 mm and 0.39 mm for AC-PC. In the Z-direction, minimal mean errors and SD were 1.07 mm and 0.93 mm on both sides with no fiducials defining Z-axial stretch. The minimal mean errors when locating RN were 0.32 mm, 0.51 mm, 1.07 mm(X, Y, Z) with SD of 0.39 mm, 0.37 mm, 0.93 mm in the right and 0.38 mm, 0.49 mm, 1.07 mm(X, Y, Z) with SD of 0.29 mm, 0.37 mm, 0.93 mm in the left, using no fiducials to define the atlas transformation.

### Coordinate Value Differences of RN and Fiducials between MRI and CVH

There were no significant differences of RN in X- and Y-direction (P>0.05) between MRI and CVH (see Table 5). But the mean coordinate values of RN in Z-direction and all the fiducials coordinates (except OX in Y-direction) between MRI and CVH is distinctly different (P<0.001).

## Discussion

To date, the best method for STN localization remains uncertain [6], [21]. Although some researchers suggest that the atlas-based methods are inaccurate because of the individual variation in positioning the STN in deep brain [22], [23], [24], others argue that STN could be identified by using standard atlas coordinates without any obvious loss in accuracy [3], [13], [21], [25].

The intention of this research was to verify the feasibility of CVH head datasets as brain atlas when positioning STN and find the appropriate structural fiducials for MRI-to-CVH local registration. Based on the research results, a patient’s head MRI can be registered to CVH for locating RN, which is adjacent to STN, with a small mean error. And the average coordinate values of gravity centers and ranges of STN in 4 CVH head datasets can be considered as references for positioning STN in patient’s head. This new approach may permit accurate and convenient identification of the location of STN before deep brain stimulation (DBS).

### The Purpose of using CVH Datasets as Brain Atlas for Chinese

Tang et al. [19] explored the differences of general features and local brain structures between Chinese brain and White brain using Tensor-based Morphology (TBM). A total of 35 cases of 3D-structure brain MRI data of Chinese male volunteers and 35 healthy Whites male subjects from the ICBM (International Consortium for Brain Mapping) datasets were included. They found that the differences in shape and size of human brain between the Chinese and White were significant (P<0.01), including the differences in mean values of length, width, height and AC–PC line distance. In addition, the White brain is a bit longer but the Chinese brain is a bit more round. The differences in 15 local structures of the cerebrum between Chinese and White are significant (p<0.01), including sulci and gyri in frontal lobe, parietal lobe, occipital lobe, temporal lobe, insular lobe, hippocampus and cingulum. Furthermore, the structures in deep brain, such as caudate and putamen were also different between the Chinese and White (p<0.01).

It is generally known that the structures in deep brain, such as caudate and putamen, are near the STN. Therefore, it is reasonable to presume that there may be some differences in STN structure between the Chinese and White, and it maybe more accurate to use the datasets, which are based on the cadaver specimens of Chinese, such as CVH.

In order to reduce individual differences and enhance the representation of the position of STN in CVH datasets, we determined the mean values of fiducials in four CVH datasets for data analysis as well as the gravity center coordinate values and ranges of STN in the X-, Y-, and Z-direction.

### The Relative Location Relationships between RN and Fiducials

All recognizable fiducials used in this research are near STN. RN is considered a reliable validation structure for STN localization because it is adjacent to STN, which lacks clarity in MRI [3]. The contours of RN remain clearly visible and regular and are easy to position for gravity center in the axial and coronal MRI sequences.

Coordinate value of the RN gravity center positively correlates with the PU-A, the PU-L and the width of the third ventricle, and negatively correlates with the patients’ age (Table 3). This result coincides with brain morphology and the principle of brain development and shrinkage. The coordinates of the RN gravity center are totally unrelated to the fiducials, AC-PC, OX in Y-direction and OX in Z-direction, which are near the RN and STN, as well as the patients’ age. This result validates that the positions of the RN gravity center, relative to the origin of coordinate system, are not affected by changes of fiducials in Y- and Z-direction and the age. It should be pointed out that the changes in fiducials coordinates reflect to some extent the differences in general or local morphology of cerebrums and midbrains among individuals. And it can be concluded that the coordinates of gravity center of RN in Y- and Z-direction have little to do with the general or local morphology of cerebrums and midbrains.

### MRI-to-CVH Registration

After comparing different registration algorithms to position the STN and studying the visible surrounding structures that can influence the STN localization, both Duay et al. [26] and Castro et al. [27] point out that the methods using local structures are much better than those used for global registration. In addition, the fiducial-based local space registration method was considered as an effective method to transform MRI to Mai atlas for positioning structures in midbrain, and the structrues close or binding to the volume of midbrain were used as fiducials [3]. Besides, the X coordinate value of the RN gravity center positively correlates with the fiducials in X-direction in Table 3, especially the PU-A and the PU-L (P<0.001). Therefore, we registered 30 MRI sequences to CVH to test whether the fiducial-based local space registration method can be applied to transform MRI to CVH for positioning RN and whether the coordinate value of RN iss automatically scaled with fiducials PU-A and PU-L in X-direction.

After registering, the general and local morphology of cerebrum and midbrain in MRI were supposed to be closer to CVH to some extent, but we obtained the result that the minimum mean errors for locating RN occur when no fiducials are used. The mean errors in the X and Y-direction are at the sub-millimeter level or nearly sub-millimeter level in the Z-direction (see Table 4). The registration quality between MRI and CVH is little affected by omitting stretch in the X-, Y- and Z-direction. Therefore, the fiducial-based local space registration method is not fit for transforming MRI to CVH for positioning RN. Altough the X coordinate value of the RN positively correlates with the PU-A and PU-L (see Table 3), it is not changed in equal proportion according to the X coordinates of PU-A and PU-L. And the differences of X coordinates of RNs in individuals are much smaller than the fiduals. These results suggest that the position of RN, relative to the origin of coordinate system (midpoint of AC-PC), can be considered as minor differences in different brains. Due to the adjacent positions between STN and RN in deep brain, we concluded that the CVH should be used as standard atlas directly to locate STN.

Videen T O et al. [3] registered 29 cases of MRI to Mai atlas using fiducials near the STN and proposed that registration quality was little affected by omitting stretch in the X-direction, but it was still considered necessary to locate AC-PC in the Y-direction and OX in the Z-direction. This result is inconsistent with our work. The differences are probably mainly due to the Mai atlas based on postmortem brain and the imprecise amount of tissue deformation in the atlas brain [3]. However, the CVH head datasets are obtained by low-temperature milling and digital photographing for integrated cadavers. During the whole processes, no deformation and distortion from brain extraction or section slicing, mounting or staining occur. Therefore, the morphologies of all head structures in CVH head datasets are closer to the living human brains than the traditional brain atlases.

### The Feasibility of CVH as Brain Atlas for STN Localization

To further verify the feasibility of CVH as brain atlas for STN localization, we calculated and analyzed the mean coordinate value differences of RN and fiducials in X-, Y- and Z-direction between 30 MRI and CVH (see Table 5).

The differences between corresponding fiducials in MRI and CVH indicated that it is impossible for CVH to reflect the whole condition of the general and local morphology of cerebrum and midbrain in MRI. However, the standard deviations(SD) of X and Y cordiantes of RN in 30 MRI are low, and no significant difference exists in X and Y cordiantes of RN between MRI and CVH (P>0.05). This result shows that the X and Y cordiantes of RN in CVH for 30 MRI are more representative. Since the RN is adjacent to STN, the ranges and gravity center coordinates of STN in CVH can be used to locate STN in MRI sequences in X- and Y-direction. The SD of Z coordinate of RN in 30 MRI is high, and the difference in Z-direction of RN between MRI sequences and CVH is significant. Therefore, the STN ranges and gravity center coordinates of CVH in Z-direction are not available for locating STN in MRI sequences directly. This may be because of the markedly individual difference in Z-direction, and the number of CVH cases is too small to represent the whole condition. However, McClelland III S et al. [21] studied the preoperative and postoperative MRI of 26 consecutive patients based on the stereotactic atlas localization, and found the effective range of the DBS electrode to be within a 6-mm-diameter cylinder centered in the presumed middle of STN. And satisfactory placement of DBS microelectrode along Z-direction seems to be less problematic during the actual procedure of STN-DBS [6]. The displacement in Z-direction can be compensated by the multiple stimulating points attached to the microelectrode and choosing the one which can obtain optimal therapeutic effect.

### Conclusions

In this study, we develop a new atlas-based approach for STN localization based on four CVH head datasets and assess its feasibility. Using CVH head datasets as standard brain atlas, we are able to localize RN, which is adjacent to STN, without reliance on subjective methods. Meanwhile, we confirmed the average ranges and coordinate values of STN in the four CVH head datasets. These results may have further implications for the STN localization during DBS, especially for Chinese PD patients. With the increasing number of CVH datasets in the future, the representation of the localization of STN will also be on the rise.
